# Supplementary material for: Hot spot prediction in protein-protein interactions by an ensemble system
Source: BMC Syst Biol. 2018 Dec 31;12(Suppl 9):132. doi: 10.1186/s12918-018-0665-8 (PMC6311905; doi:10.1186/s12918-018-0665-8)
Supplement: Supplementary file 2 — All relevant MATLAB code. A simple Matlab implement of our predictor. (ZIP 19,742 kb) [file 12918_2018_665_MOESM2_ESM.zip › Additional file 2/Read ASA/ASA Property/BID/1LQBD.txt]

 NetSurfP - Protein Surface Accessibility and Secondary Structure Predictions

  

|  |  |  |
| --- | --- | --- |
|  |  | NetSurfP - Protein Surface Accessibility and Secondary Structure Predictions  Technical University of Denmark |

  


---

```
# For publication of results, please cite:
# A generic method for assignment of reliability scores applied to solvent accessibility predictions.
# Bent Petersen, Thomas Nordahl Petersen, Pernille Andersen, Morten Nielsen and Claus Lundegaard
# BMC Structural Biology 2009, 9:51 doi:10.1186/1472-6807-9-51
#
# Column 1: Class assignment - B for buried or E for Exposed - Threshold: 25% exposure, but not based on RSA
# Column 2: Amino acid
# Column 3: Sequence name
# Column 4: Amino acid number
# Column 5: Relative Surface Accessibility - RSA
# Column 6: Absolute Surface Accessibility
# Column 7: Z-fit score for RSA prediction
# Column 8: Probability for Alpha-Helix
# Column 9: Probability for Beta-strand
# Column 10: Probability for Coil
E P  1LQBD                  1    0.755 107.120  -0.364   0.003   0.003   0.994
E F  1LQBD                  2    0.329  66.010  -2.456   0.018   0.047   0.935
E S  1LQBD                  3    0.531  62.186  -1.025   0.018   0.047   0.935
E T  1LQBD                  4    0.387  53.635  -1.122   0.018   0.088   0.893
E Q  1LQBD                  5    0.589 105.160  -0.129   0.053   0.043   0.903
E D  1LQBD                  6    0.393  56.602  -1.496   0.053   0.043   0.903
E T  1LQBD                  7    0.522  72.443  -0.559   0.056   0.142   0.802
E D  1LQBD                  8    0.429  61.790  -1.117   0.118   0.150   0.732
B L  1LQBD                  9    0.087  15.875   0.641   0.118   0.150   0.732
E D  1LQBD                 10    0.258  37.178   0.344   0.199   0.152   0.649
B L  1LQBD                 11    0.069  12.671   0.702   0.216   0.235   0.548
E E  1LQBD                 12    0.353  61.652   0.318   0.231   0.330   0.439
B M  1LQBD                 13    0.129  25.733   0.134   0.231   0.330   0.439
B L  1LQBD                 14    0.044   8.111   0.063   0.231   0.330   0.439
B A  1LQBD                 15    0.076   8.386  -0.430   0.064   0.216   0.721
B P  1LQBD                 16    0.174  24.620  -1.469   0.056   0.142   0.802
B Y  1LQBD                 17    0.176  37.568  -0.827   0.020   0.205   0.775
B I  1LQBD                 18    0.051   9.379   0.732   0.020   0.205   0.775
B P  1LQBD                 19    0.202  28.635  -0.398   0.019   0.141   0.840
B M  1LQBD                 20    0.147  29.455  -0.333   0.018   0.088   0.893
E D  1LQBD                 21    0.349  50.305  -0.727   0.018   0.047   0.935
E D  1LQBD                 22    0.392  56.530  -1.080   0.053   0.043   0.903
E D  1LQBD                 23    0.306  44.123  -0.945   0.052   0.084   0.864
B F  1LQBD                 24    0.072  14.450   0.181   0.064   0.216   0.721
E Q  1LQBD                 25    0.309  55.187   0.586   0.022   0.552   0.426
B L  1LQBD                 26    0.077  14.044  -0.304   0.023   0.655   0.322
E R  1LQBD                 27    0.292  66.914   0.337   0.022   0.552   0.426
E S  1LQBD                 28    0.302  35.359  -0.871   0.022   0.359   0.619
B F  1LQBD                 29    0.156  31.309   0.027   0.019   0.141   0.840
E D  1LQBD                 30    0.399  57.424  -0.285   0.018   0.047   0.935
E Q  1LQBD                 31    0.514  91.783  -1.363   0.018   0.047   0.935
E L  1LQBD                 32    0.537  98.270  -2.788   0.018   0.047   0.935
E S  1LQBD                 33    0.597  69.933  -1.286   0.005   0.015   0.979
E P  1LQBD                 34    0.806 114.442  -1.944   0.003   0.003   0.994

Explain the output.  Go back.
```

---
